# Nutrient Requirements and Fertilizer Management Strategies in Plant Cultivation

**DOI:** 10.3390/plants14192981

**Published:** 2025-09-26

**Authors:** Lin Tang

**Affiliations:** Department of Agroecology, Aarhus University, 8830 Tjele, Denmark; lin.tang@agro.au.dk; Tel.: +45-50108877

## 1. Introduction

Essential plant nutrients are fundamental to plant growth and reproduction, and deficiencies in any of them can disrupt or even halt the plant growth cycle [1]. In cultivated systems, it is often necessary to supplement soil fertility through the judicious application of fertilizers in order to meet crop nutrient demands and maintain or increase yields [2,3]. Accurate nutrient diagnosis is a key step in determining plant nutritional status and provides crucial guidance for allocating fertilizers to producers and advisors [4,5].

Balanced fertilization refers to supplying essential nutrients in optimal quantities and appropriate proportions, using suitable application methods and timing to match the specific requirements of crop needs and agroclimatic conditions [6,7]. Developing novel and sophisticated fertilization strategies remains a major challenge in nutrient management [8,9]. Optimized nutrient management not only prevents deficiencies, imbalances or excessive fertilizer use, but also enhances nutrient-use efficiency, supports productive agronomic systems and promotes environmentally sustainable plant cultivation [10,11].

In such a context, this Special Issue (SI) aims at advancing our understanding of nutrient requirements and fertilizer management strategies in plant cultivation. It considers not only the direct impacts of fertilizer practices on nutrient uptake and utilization, but also the indirect effects of water management and soil improvement on nutrient dynamics. The SI comprises eight original research articles contributed by authors from institutions in China, Italy, Mexico and the United States. Together, these studies provide an integrated perspective on a wide range of diverse plant species, including legumes, cereals, fruits, medicinal herbs, trees and sugar crops cultivated in diverse systems such as hydroponics, substrate culture, pot experiments and field trials, and examined across physiological, molecular and ecological scales under different climatic conditions.

## 2. Advances in Nutrient Requirements and Fertilizer Management Strategies in Plant Cultivation

The following section presents an overview of the articles included in this SI, highlighting how their findings collectively advance research on nutrient requirements and fertilizer management strategies in plant cultivation.

Shohag et al. [12] conducted a two-year field experiment to evaluate the combined effects of phosphorus (P) and oxygen (O_2_) fertilization on plant growth, pod yield and P uptake of snap bean (*Phaseolus vulgaris* L.) grown in acidic sandy soil. Their study showed that applying triple superphosphate together with calcium peroxide significantly enhanced vegetative growth and pod production. Additionally, strong associations were observed between growth, pod yield and nutrient accumulation, and notable seasonal differences emerged in both crop performance and soil P dynamics. This research highlights that incorporating O_2_ fertilizers is a cost-effective strategy to alleviate hypoxic stress, improve P use efficiency and increase yields in snap bean cultivation.

Cheng et al. [13] investigated the effects of a soil amendment (SA) on agronomic performance, seed nutritional quality and cadmium (Cd) accumulation in several faba bean (*Vicia faba* L.) genotypes cultivated in acidic soils. Their study showed that SA application raised soil pH, lowered phytoavailable Cd and reduced Cd concentrations in seeds while alleviating soil manganese and aluminum toxicity. In addition, SA enhanced the levels of essential macronutrients and micronutrients in seeds, while reducing phytate content, with only a marginal decrease in average yield. This research provides valuable evidence of the potential of SA to improve nutrient fortification and mitigate Cd contamination in farmland affected by Cd pollution.

Yu et al. [14] conducted a series of pot experiments to exam how soil microbial P mobilization and rice P uptake respond to applications of rice husk biochar immobilized with *Bacillus megaterium* (BMB). Their results revealed that the treatment combining organic fertilizer with BMB (MOF) produced the most pronounced improvements in soil available P and fostered beneficial shifts in bacterial community structure, thereby enlarging the pool of bioavailable P reservoir. Moreover, the MOF treatment significantly enhanced soil carbon utilization and rice agronomic performance. This research offers a valuable theoretical basis for understanding the role of biochar-based bacterial inoculants in improving P management in rice systems.

Raffaelli et al. [15] conducted a two-year soilless greenhouse experiment to identify strawberry (*Fragaria × ananassa*) genotypes with superior capacity to sustain yield and enhance fruit quality under reduced irrigation. Their findings showed that moderate water restriction improved several quality attributes, including citric acid concentration, soluble solids content and color brightness, while fruit firmness remained unaffected. Total phenolic content exhibited the strongest response to water stress, indicating enhanced antioxidant activity. This research underscores the importance of selecting genotypes best suited to water regimes in order to enhance fruit quality, maintain high production standards and promote water-use efficiency in strawberry cultivation.

Peng et al. [16] conducted a three-year field experiment to quantify the effects of tillage methods prior to wheat sowing and irrigation practices during the wheat season on the yield formation and water-use efficiency of subsequent summer maize production. Their results showed that, compared with rotary tillage and conventional plowing, subsoiling before wheat sowing significantly increased soil water storage at maize sowing, thereby enhancing dry matter accumulation and nutrient uptake in maize. One-off irrigation during the wheat season negatively affected pre-sowing soil water storage and maize productivity; subsoiling effectively mitigates these adverse effects. Overall, tillage practices before wheat sowing exerted a greater influence on soil water storage and maize quality than irrigation strategies applied during the wheat growing season. This research provides practical guidance for optimizing soil water use and improving maize productivity in dryland systems.

Flores-Sánchez et al. [17] investigated nutrient uptake dynamics in two *Jaltomata* species grown under three levels of electrical conductivity in greenhouse hydroponic systems. Their study revealed marked variability in nutrient concentration and total dry matter (TDM) accumulation between species and across phenological stages. For macronutrients, *J. procubens* showed the highest P concentrations from the vegetative stage, magnesium up to fructification, potassium during the vegetative phase and nitrogen (N) at flowing, whereas *J. tlaxcala* accumulated more calcium during fructification. Regarding micronutrients, *J. thaxcala* exhibited greater iron uptake from the vegetative stage, higher boron, zinc and TDM during flowering, and manganese at fructification. This research suggests that tailored fertilization programs are necessary for each species to optimize nutrient supply and productivity.

Wang et al. [18] carried out a six-year field study to evaluate the long-term effects of drip irrigation and N application on growth, biomass allocation and the efficiency of water and fertilizer use in short-rotation triploid *Populus tomentosa* plantations. Their results indicated that the impacts of water and N treatments on the annual increment of diameter at breast height, tree height, stand basal area (BA_S_), stand volume (V_S_) and annual forest productivity (AFP) were strongly influenced by stand age. Moreover, irrigation significantly increased BA_S_, V_S_, AFP and total forest biomass of six-year-old stands, with irrigation exerting a more consistent effect than N fertilization. This research provides valuable guidance for optimizing water and fertilizer management in short-rotation poplar plantations.

Wang et al. [19] conducted a three-year field experiment to assess the effects of irrigation levels and N application rates on sugar yield, N use efficiency and soil nitrate-N (NO_3_^−^-N) residues in drip-fertigated sugar beet cultivated in an arid region of China. Their results showed that sugar yield and N uptake increased and then plateaued with high N rate, whereas N use efficiency decreased. Greater irrigation volumes reduced residual NO_3_^−^-N in soil profiles. Based on these findings, the authors developed a reliable N nutrition index for sugar yield, derived from a critical N dilution curve established from sugar beet dry matter. This research provides an important basis for improving water and N management in sugar beet production across arid and semi-arid regions worldwide.

## 3. Conclusions

In summary, this SI highlights the rapidly advancing scientific frontiers of nutrient requirements and fertilizer management strategies in plant cultivation. It brings together current research findings and provides valuable theoretical foundations as well as practical pathways for addressing the increasingly complex challenges in this field. As the Guest Editor, I am deeply grateful to all the authors for contributing their high-quality research to this SI and to the reviewers for their careful assessments, which ensured the scientific rigor of the published papers. I hope that the articles presented here will serve as a rich source of knowledge, inspiration and fresh perspectives on nutrient requirements and fertilizer management strategies in plant cultivation for both researchers and practitioners.
